# Supplementary material for: Are carbon emissions trading and green financial instruments synergistic? -Comprehensive quantitative research based on content analysis
Source: PLoS One. 2024 Mar 7;19(3):e0298601. doi: 10.1371/journal.pone.0298601 (PMC10919601; doi:10.1371/journal.pone.0298601)
Supplement: S1 Data — (ZIP) [file pone.0298601.s001.zip › Data/地级市Z_F工作报告.zip!/╦─┤¿/╤┼░▓╩╨/└┤╫╘╣1⁄2╓┌║┼í╛┬φ┐╦╩2╛▌═°í┐_2014.docx]

各位代表：

　　现在，我代表市人民政府，向大会作政府工作报告，请予审查，并请市政协各位委员提出意见。

　　万众一心、顽强拼搏 “4·20”芦山强烈地震抗震救灾取得阶段性重大胜利

　　2013年4月20日，芦山县发生7.0级地震，这是我市有史以来破坏性最强、波及范围最广、救灾难度最大的一次自然灾害，是雅安五年内遭受的第二次大地震。全市8县(区)全部受灾，176人遇难，城乡居民住房、基础设施、公共服务设施、三次产业遭受重创，生态环境遭受严重破坏。面对突如其来的地震灾害，国家、省、市第一时间启动地震ⅰ级应急响应。党中央、国务院与灾区人民心心相连，习近平总书记、李克强总理、张高丽副总理等党和国家领导人亲临灾区，看望慰问干部群众和救援人员，指导抗震救灾。省委、省政府在芦山成立抗震救灾前线指挥部，王东明书记、魏宏省长亲自指挥。人民解放军指战员、武警部队官兵、民兵预备役人员、公安民警、专业救援人员以及医疗卫生工作者、新闻工作者、志愿者等，从全国四面八方驰援雅安。市委、市政府紧急动员、精心部署，团结带领全市广大干部群众，万众一心、顽强拼搏，夺取了抗震救灾阶段性重大胜利。

　　争分夺秒抢险救援。坚持生命至上，第一时间组织广大干部群众自救互救，统筹市内各种救援力量全力搜救被困群众、救治受伤人员。抢通因灾断道的国省干线公路和通乡通村公路，保证生命通道畅通。全力做好保障和协调工作，确保成都军区、成都军区空军、省军区、武警四川省总队等救援队伍，以及社会各界力量，迅速投入抢险救援。累计从废墟中搜救生还者163人，解救被困群众1800多人，救治伤员3万多人次，转移救治重伤员426人。同步开展卫生防疫工作，确保了大灾之后无大疫。

　　妥善安置受灾群众。紧急转移、应急安置受灾群众71万余人。搭建帐篷11.7万顶，安装活动板房13.3万平方米，农村自建过渡房68839户，震后3个月全部完成受灾群众过渡安置。加强救灾资金和物资保障，调度应急救灾综合补助资金17亿元，及时发放各类救灾物资4.6万吨。落实各项救助政策，发放救灾口粮3.19万吨、生活救助金7.05亿元、过渡安置费2.32亿元，确保了受灾群众有饭吃、有衣穿、有干净水喝、有临时住处、有医疗救助。

　　迅速恢复生产生活。全力抢修受损基础设施和公共服务设施，确保群众生产生活需要。震后6小时，芦山恢复金融服务。震后9小时，雨城、名山恢复供气。震后36小时，全市商业网点基本恢复营运。震后7天，灾区通信全部恢复。震后10天，灾区基本恢复正常供水。震后15天，全市中小学全面实现复课。震后20天，灾区基本恢复正常供电。全面排查整治企业安全隐患，积极帮助企业恢复生产，震后1个月，受灾工业企业复产率达到50%以上。

　　强力推进灾后恢复重建。配合编制《芦山地震灾后恢复重建总体规划》和11个专项规划。全市2251个项目纳入总体规划，估算总投资764.4亿元。坚守“五位一体”工作底线，加快灾后恢复重建项目建设，截至去年底，开工项目564个，开工率25.1%，完成投资115亿元，占估算总投资的15%。始终把城乡居民住房重建作为恢复重建的首要任务，在确保质量和安全前提下加快推进。落实灾损住房维修加固和重建补助政策，及时兑现补助资金19.64亿元。设立农房重建贷款担保基金，实施贷款贴息和利率优惠叠加政策，帮助重建农户贷款建房，发放贷款9.3亿元。加强建材供应和交通运输等保障。农房维修加固全面完成，农房重建开工5.7万户、开工率70.7%;城镇住房重建开工1.8万套、开工率46.1%。坚持优先安排学校、医院等公共服务设施恢复重建，212所学校维修加固全面完成，学校新建项目开工43个，医疗卫生类项目开工43个，就业和社会保障、文化体育、社会管理等公共服务设施恢复重建有序推进。加快推进以交通为龙头的重大基础设施建设，国道351线土建工程、国道318线和108线改造工程开工建设。把地质灾害防治作为“生命工程”，全面落实监测、预警、排险、避让等措施，完成排危除险项目275个，启动工程治理项目30个，宝兴县冷木沟、教场沟等重大地质灾害应急治理工程加快推进。强化协调服务，对口援建项目开工8个。

　　扎实有效开展“暖冬行动”。去年入冬以来，我们切实把受灾群众安全温暖过冬作为灾区的头等民生大事，严格落实“四防两保两加强”措施，对11738户群众自建过渡安置房进行保温加固，625户特困户实现搬新家过新年，4482户困难群众建房得到“一对一”帮扶;发放棉被49853床、棉衣裤28615件、取暖用品8085件、粮油221.8吨，成功实现“不遗漏一户、不遗漏一人，没有一人挨饿受冻”的目标。

　　各位代表，“4·20”芦山强烈地震，既是一次重大磨难，也是一次重大考验。面对灾难，我们万众一心、顽强拼搏，用理想凝聚力量，用信念铸就坚强，用真情凝结关爱，最大限度地挽救了受灾群众生命，最大限度地降低了灾害造成的损失，灾区社会秩序迅速恢复正常，受灾群众得到妥善安置，灾后恢复重建迅速开创新局面。取得这样的成绩，得益于社会主义制度的巨大优越性，得益于党中央、国务院，省委、省政府的坚强领导，得益于中央各部委、省级各部门、兄弟市(州)的倾力支持，得益于全国人民的无私援助，得益于人民解放军指战员、武警部队官兵的英勇奋战，得益于全市广大干部群众的顽强拼搏。在此，我代表市人民政府，向奋勇投身我市抗震救灾的广大干部群众，人民解放军指战员、武警部队官兵、民兵预备役人员、公安民警，广大医疗卫生工作者、工程建设者，广大志愿者和新闻工作者，致以崇高的敬意!向倾力支援我市抗震救灾的中央各部委、省级各部门、兄弟市(州)和社会各界，向德阳、绵阳、南充、攀枝花、自贡、泸州6个援建市，向踊跃为灾区提供援助的港澳台同胞、海外华侨华人和国际社会、国际友人，表示衷心的感谢!

　　凝心聚力、克难奋进 2013年经济社会实现平稳发展

　　过去一年，在省委、省政府和市委的坚强领导下，在市人大、市政协的监督支持下，市人民政府认真履行职能，紧紧依靠全市各族人民，沉着应对多重困难挑战，在全力抗震救灾的同时，实现了经济社会的平稳发展。

　　主要经济指标止滑回升。受“4·20”芦山强烈地震重大自然灾害影响，全市经济发展呈现明显的阶段性特征。第一季度，经济发展呈现良好态势，地区生产总值增长10.7%。地震发生后，经济发展严重受挫，上半年地区生产总值仅增长1.2%。面对严峻形势，我们全力组织恢复生产，主要经济指标逐步回升。全年实现地区生产总值417.97亿元，增长3.9%;地方公共财政收入22.94亿元，完成调整预算的104.27%，下降24.09%;全社会固定资产投资352.82亿元，下降1.2%;社会消费品零售总额146.32亿元，增长10.9%;城镇居民人均可支配收入22254元，增长11%;农民人均纯收入8093元，增长12.6%;居民消费价格上涨2.9%。

　　三次产业恢复发展。第一产业增加值增长2.1%。粮食产量达到46.4万吨。新增特色产业基地8.8万亩、市级以上农业产业化龙头企业7户、国家地理标志保护产品3个、有机农畜产品认证27个、四川省著名商标4件。新增农民专业合作社394个。转移农村劳动力34.9万人，实现劳务收入38.3亿元。第二产业增加值增长3.2%，其中规模以上工业增加值增长3.5%。主营业务收入上亿元企业达到84户，新增规模以上企业17户。“3+1”优势产业实现产值350.3亿元，占全市规模以上工业总产值的85.7%。一名微晶、王老吉等产业项目加快推进。芦天宝飞地产业园区(四川雅安经济开发区)、芦山产业集中区、荥经循环经济示范园区加快建设。建成投运7个110千伏及以上输变电工程。第三产业增加值增长6.4%。实现旅游综合收入70.7亿元，旅游从业人员达到2.4万人;新增国家4a级旅游景区2个。新开工商品房面积84.45万平方米，竣工面积31.77万平方米。金融、保险、运输、通讯等服务业加快发展。

　　城乡面貌不断改善。主城区雅州大道、雅园路、青年路实现改造提升;水中坝安置小区、大兴公租房等项目开工建设;老城区10.5千米供水管网改造完成，15处污水截流工程全面完成;南郊备用水源工程投入试运行。名山区和各县城建设加快推进。重点集镇建设步伐加快，荥经县龙苍沟镇、汉源县九襄镇全面启动“全省百镇建设试点行动”。将灾后恢复重建和新农村建设有机结合，启动建设省级新农村成片推进示范县3个;规划的237个灾后新村聚居点开工建设193个。 乐雅高速公路建成通车。四川省环境优美示范城市创建成功，省级园林城市创建通过考评验收，国家级卫生城市、国家智慧城市和省级双拥模范城市创建步伐加快。

　　社会事业协调发展。组织实施科技项目98个，高新技术产业产值突破50亿元。“两免一补”工程扶助学生28.69万人次，营养改善计划惠及10.27万名农村义务教育学生，资助普通高中家庭经济困难学生6543人，开工建设幼儿园19个，6所普通高中达到教育现代化标准，高考录取率76.2%。乡村卫生一体化加快推进，公共卫生服务网络更加完善;在全省率先发放居民健康卡;9家县级及以上医院加快创建四川省数字化医院。建成省级示范乡镇综合文化站16个，公共文化服务单位全部免费开放。新建全民健身路径64条，陆家坝体育活动中心开工建设。深入开展“六五”普法。有效排查化解社会矛盾，集中解决了一批关系群众利益、影响社会稳定的突出问题。严密防范和打击各类违法犯罪活动，社会治安平稳有序。强化安全生产，开展食品药品、矿山、交通、消防等重点行业和领域专项整治，遏制了重特大事故发生。市级综合应急指挥平台初步建成。人口自然增长率3.54‰。民族、宗教、防震减灾、档案、保密、科协、地方志、社会科学、国防动员和民兵预备役等工作扎实推进。

　　社会保障全面加强。公共财政支出中民生支出171.5亿元，占65.71%。“十项民生工程”全面完成。城镇新增就业2.23万人，城镇登记失业率3.89%，发放创业小额担保贷款7092万元。城市居民最低生活保障标准提高到每人每月320元，农村居民最低生活保障标准提高到每人每年1800元。城乡居民养老保险覆盖46.4万人。“五保”对象集中供养率达到61.15%。新农合参合率达到99.6%，城镇居民医保参保率达到96%。城乡医疗救助14.7万人次。扶持17210名农村扶贫对象改善生产生活条件。保障性住房完成8083套。妇女、儿童、老龄、残疾人、慈善、红十字等工作扎实推进。

　　改革开放步伐加快。行政审批事项下放195项，行政审批项目承诺办理时限在法定时限基础上提速62.5%。雅安市商业银行增资扩股工作全面启动，雅安农村商业银行加快筹建。国有企业改革稳步推进。“营改增”试点顺利推进。水、电、气等资源性产品价格改革启动实施。食品药品监管体制、医药卫生体制、林权制度、户籍制度等改革深入推进。与成都、凉山、攀枝花等周边市(州)的区域合作不断深化。汉源县、石棉县纳入攀西战略资源创新开发试验区并启动相关工作。石棉县独立工矿区转型发展试点城市建设加快推进。积极参加“三大招商活动”和第十四届西博会等大型会展活动，到位市外资金254.4亿元，增长9.65%。实现进出口总额6887万美元，增长61.33%;实际利用外资1142万美元。外事、侨务、对台工作取得新进展。

　　自然生态和环境保护持续加强。全国生态文明示范工程试点工作有序推进。森林覆盖率达到62.7%。管护天然林1296.06万亩，巩固退耕还林成果94.27万亩，完成营造林48.32万亩。综合治理水土流失面积214.7平方公里。建成省级生态乡镇5个、生态村47个。栗子坪自然保护区晋升为国家级自然保护区，并成功放归第3只大熊猫。淘汰落后生产能力企业19户。万元地区生产总值能耗下降3.42%，万元工业增加值能耗下降7.16%。

　　在抓好经济社会发展的同时，我们更加注重政府自身建设。自觉接受市人大及其常委会的法律监督、工作监督和市政协的民主监督，虚心接受群众监督和社会舆论监督，主动办理人大代表建议、批评、意见152件和政协委员提案225件，办结率100%。认真落实政府信息公开条例，政务公开工作深入推进。强化政风行风建设，严格遵守中央“八项规定”，财政一般性支出压减2423万元。行政监察和审计监督工作深入推进。

　　各位代表，大灾之年，全市经济社会发展取得这样的成绩实属不易。这是在党中央、国务院，省委、省政府的坚强领导和亲切关怀下，市委统揽全局、精心部署的结果，是市人大、市政协和人大代表、政协委员监督支持的结果，是全市广大干部群众共同奋斗的结果。过去一年，全市人民戮力同心、共克时艰，汇聚了无穷的信心与力量;各级干部呕心沥血、无私奉献，付出了超常的艰辛与努力;广大官兵赤胆忠心、直面艰险，奉献了无悔的热血与生命;各界人士肝胆相照、同舟共济，倾注了无尽的爱心与真情。在此，我代表市人民政府，向全市人民，向支持和参与雅安建设的各界人士，表示衷心的感谢，并致以崇高的敬意!

　　在看到成绩的同时，我们也清醒地认识到，在发展和工作中还存在不少的问题。由于“4·20”芦山强烈地震影响，去年经济发展的部分预期目标没有实现。灾后恢复重建任务艰巨繁重，特别是项目建设和产业重建速度亟待进一步加快。工业总量小、水平低，产业链条短，园区承载能力弱，调结构、转方式任务艰巨;现代农业基础薄弱，农业产业化龙头企业亟需加快培育;高品质的旅游开发不足，现代服务业发展滞后。招商引资质量亟待提升。城镇化步伐缓慢，城镇建设和管理力度需进一步加大。教育事业发展还有较大差距，老百姓对优质教育的渴望在短期内难以满足。政风行风还需进一步改进。我们将诚恳接受人民群众和各位代表的批评，以敢于亮丑的勇气查找问题，以勇于担责的态度剖析原因，以勤于补短的行动实施整改，努力把政府工作提高到一个新的水平。

　　抢抓机遇、奋勇攀登 2014年各项工作务求新成效

　　2014年是雅安灾后恢复重建具有决定性意义的一年，也是在新的起点上建设幸福美丽新家园的开局之年。挑战前所未有，机遇千载难逢。今年政府工作的基本思路是：深入贯彻党的十八大和十八届二中、三中全会，中央经济工作会议和城镇化工作会议精神，认真落实省委十届三次、四次全会，省委经济工作暨城镇化工作会议精神，坚决执行市委三届七次全会的决策部署，围绕后发追赶战略构想，将生态文明建设融入经济社会发展全领域、全过程，以科学发展、加快发展为主题，以灾后恢复重建为主线，以改革开放为动力，以增进民生福祉为目标，坚持“打硬仗”、“补欠账”，抓住灾后恢复重建大机遇，做好生态文明示范大文章，建设幸福美丽新家园。

今年全市经济社会发展的主要预期目标是：地区生产总值增长11%;全社会固定资产投资增长27.5%;社会消费品零售总额增长11.5%;地方公共财政收入增长11%;城镇居民人均可支配收入增长10%，农民人均纯收入增长11%;居民消费价格涨幅控制在3.5%左右;城镇登记失业率控制在4%以内;人口自然增长率控制在5‰以内;城镇化率提高1.5个百分点;完成省上下达的节能减排任务。

为确保实现上述目标，我们将抓好十个方面的工作。

　　一、坚持民生优先，全面推进灾后恢复重建

　　灾后恢复重建是事关雅安人民福祉的民生大事，也是我市加快后发追赶的历史性机遇。

　　加快规划项目的组织实施。纳入总体规划的2251个项目，确保4月20日前全面开工、7月20日前全部开工，年底累计完工907个、完成投资456亿元，建成一批事关民生和发展的重要项目，灾后恢复重建取得关键性胜利。

　　优先实施城乡住房重建。兑现补助政策，强化信贷支持，做好建材供应、技术服务等保障工作，落实抗震设防标准，确保到3月底前，237个新村聚居点和农户建房全部开工;4月20日前，4482户由政府特别帮扶的困难群众建房全部完工，城镇住房重建全部开工;6月底前，城镇受损住房维修加固全部完工;年底农房重建全部完工，城镇住房重建完工30%以上。

　　加快公共服务设施重建。将学校、医院重建放在优先位置，抓紧开工建设，力争早日建成并投入使用。确保到4月20日前，累计开工公共服务设施项目957个，教育、卫生类重建项目全部开工;年底完工公共服务设施项目603个。

　　加快基础设施重建。以恢复和提高保障能力为重点，加快推进交通、水利等基础设施重建。强力推进3条国道、5条经济干线和农村公路建设，加快震损水库、堤防、灌溉等水利基础设施恢复重建，力争一批项目取得突破性进展。确保到4月20日前，累计开工基础设施项目95个;年底完工47个。

　　加快城镇体系重建。强化“中心带动、轴线集聚、县城提升、整体推进”，加强中心城区、县城和16个重点镇建设，优化空间布局，完善城市基础设施和公共服务设施，提升综合承载能力。确保到4月20日前，累计开工项目109个;年底完工5个。

　　加快产业重建。强化产业园区建设，积极招商引资和承接产业转移。依托优势资源发展特色产业，增强灾区经济实力和发展后劲。确保到4月20日前，累计开工产业重建项目181个;年底完工63个。

　　抓好地质灾害防治和生态修复。坚持把地质灾害防治作为“生命工程”贯穿灾后恢复重建始终，强化监测预警，抓好排危除险。主汛期前，冷木沟、教场沟等地质灾害治理工程，185处应急治理、排危除险工程，107处损毁工程全部完成;年底累计完工地质灾害防治项目38个。加快生态修复，4月20日前，累计开工项目137个;年底完工37个。

　　统筹推进农村建设、土地利用、防灾减灾等工作。

　　二、加快转型升级，强力推进新型工业化

　　推进工业强市，加快新型工业化进程，是我市迫在眉睫的任务。

　　加快园区建设发展。强化园区基础设施和配套公共服务设施建设，提升产业承载能力和服务保障水平，完善园区运营管理模式，推动工业向园区集中。高质量招商引资，积极承接产业转移。芦天宝飞地产业园区(四川雅安经济开发区)力争升级为国家级经济技术开发区。加快石棉、汉源两个省级工业园区和荥经循环经济示范园区建设。大力支持芦山产业集中区等县级工业集中区做优做强。

　　积极培育主导产业。依托自身优势，强力推进招商引资和承接产业转移，以“3+1”优势产业为基础，促进现有企业提质增效扩能，通过5年时间，努力将水电矿冶培育成为500亿元产业，农产品加工培育成为300亿元产业(其中：茶叶产业100亿元、林竹产业50亿元、中药材产业50亿元)，机械制造培育成为100亿元产业，新材料新能源培育成为100亿元产业。今年，水电矿冶产业产值突破230亿元，机械制造业产值突破60亿元，新材料新能源产业产值突破60亿元，茶叶产业综合产值突破50亿元，中药材产业综合产值突破20亿元。积极引进、培育发展生物制药、高端装备制造和新一代信息技术等产业。

　　扶持壮大骨干企业。保障骨干企业建设用地，对重点项目优先推荐国家和省上扶持计划，优先纳入金融机构信贷支持计划，优先列入招大引强推介对象。扶持中小企业发展，力争新培育销售收入过10亿元企业2户、过5亿元企业4户、过1亿元企业8户。鼓励、支持有条件的企业上市融资。加强企业家队伍建设。帮助企业引进和留住高端人才。全年新增规模以上工业企业18户。规模以上工业增加值增长11%、主营业务收入增长10%。

　　强力推进转型升级。支持企业加快建设国家和省级技术中心、工程中心、重点实验室等科研平台，着力构建以企业为主体、市场为导向、产学研相结合的技术创新体系。启动碲铋项目科技攻关工作。强化企业技术改造，提升发展铅锌、磷化工、工业硅等载能产业，纺织、化工、建材等传统产业。加快雅化集团、青江机械、金红叶等企业退城入园步伐。支持优势企业并购重组落后企业和困难企业，鼓励关联产业和上下游企业联合重组。淘汰落后生产能力企业17户以上。

　　三、坚持强农惠农，全面深化“三农”工作

　　农业发展、农村繁荣、农民增收，始终关系我市发展大局。

　　全面落实强农惠农政策。稳定家庭联产承包责任制。加大财政对“三农”工作的投入力度。及时足额兑付粮食直补、农资综合直补、农作物良种补贴、农机购置补贴等各项惠农补贴资金。做好政策性农业保险工作。整合涉农资金，打捆使用、重点投放。

　　加强农村基础设施建设。强化山水田林路等农田水利基础设施建设，修复受损农田3万亩以上;改造农村公路290公里，完成农村公路灾后恢复重建350公里;新增有效灌面4.71万亩;新建农村沼气池6200口;改善和解决30万农村人口饮水安全问题。

　　大力发展特色效益农业。加快国家绿色(有机)农业示范区建设。深入推进现代农业、畜牧业、林业产业重点县建设，继续抓好万亩亿元示范区建设。做优做强茶叶产业，发展壮大中药材、果蔬、畜牧、水产、林下种养等特色产业，培育发展林竹精深加工业。新增有机食品生产基地3万亩，改造低产茶园10万亩，新建干果产业基地5万亩。抓好粮食生产。强化农产品质量安全监管，加快绿色食品、有机食品、地理标志产品认证。

　　推进农业产业化经营。鼓励、扶持专业大户、家庭农场、农民专业合作社、农业企业等新型农业经营主体。加快建设雅安国家农业科技园区、芦山现代生态农业示范园区，加强农业科技研发和成果转化。做大做强现有省市级重点龙头企业，扶持发展一批基础好、潜力大、带动力强的成长型龙头企业。进一步扩大“蒙顶山茶”、“大渡河”、“二郎山”等品牌的知名度，全力创建一批特色农产品品牌。新增100家、规范发展200家农民专业合作社。

　　加快建设幸福美丽新村。按照“业兴、家富、人和、村美”的要求，实施“百点新村”工程，加快推进首批20个幸福美丽新村建设试点。扎实推进石棉、汉源、雨城3个第二批省级新农村示范片建设。深入推进农村人居环境整治，不断改善农村生活条件。

　　四、发挥特色优势，加快发展现代服务业

　　加快发展现代服务业，对于稳增长、调结构、扩内需、促就业具有重大的现实意义。

　　重点发展文化旅游产业。加快大熊猫文化产业园、茶马古道民族文化产业园建设，加速构建大熊猫生态文化、茶马古道历史文化等文化旅游产业带，积极建设国家生态文化旅游融合发展试验区。加快受损旅游基础设施重建，构建市内旅游环线，提升旅游综合接待服务能力。碧峰峡、蒙顶山景区要全力创建国家5a级旅游景区;加快栗子坪(孟获城)、周公山温泉、喇叭河、东拉山等景区深度开发;强力推进牛背山、汉源湖、大川河等景区招商工作。积极推进乡村旅游发展。加大藏茶、黑砂、木雕、石雕等旅游文化产品开发力度。提升旅游景区管理和服务水平。全年旅游综合收入80.5亿元，增长13.9%。

　　积极发展生产性服务业。努力构建政策性银行、股份制银行、农村商业银行等类型多样、优势互补的金融业服务体系，大力推进金融产品服务创新，鼓励金融机构向县(区)延伸发展，规范民间融资活动。启动建设雅安物流园区，加快建设川滇藏商贸物流园区、天全物流中心。启动建设汽车零配件、特色农产品等专业市场。加快宽带通信网、数字电视网和互联网“三网”融合，推进4g网络建设和应用，推进云计算数据中心建设。引导、支持发展中介服务机构和专业商务服务机构。

　　加快发展生活性服务业。启动建设城市商贸中心，加快农贸市场建设。强化产销对接和农超对接，推进农村商业连锁经营和统一配送。抓好电子商务推广应用。加快实施“民芯工程”。支持养老、健康、文体娱乐等产业发展，加快推进养老、失业、医疗、社会福利等社会保障、管理和服务向社区延伸。引导特色餐饮业转型发展。

　　五、强化投资拉动，加快推进重大项目建设

　　项目投资既能增加即期需求，又能扩大未来供给，在相当长时间内都是我市经济工作的重要抓手。

　　加快建设一批项目。实施市以上重大项目391个，完成投资243亿元。加快推进川藏铁路成都(朝阳湖)至雅安段、雅康高速公路等重大交通项目建设;积极推进铜头引水、永定桥水库、九龙水库等水利项目建设;加快实施大岗山、锅浪跷、民治、出居沟等能源项目;开工建设雅安至武汉1000千伏特高压输变电工程，加快推进110千伏及以上输变电工程建设。

　　储备引进一批项目。深入研究国家产业政策和投资方向，从重建政策、对口援建、优势资源、产业配套等方面策划挖掘项目，争取国家和省上支持，补充重点项目储备库。着力引进一批投资量大、带动力强的先进制造业和服务业项目，提高产业性投资占全社会固定资产投资的比重，不断增强发展后劲。

　　强化项目要素保障。完善领导联系重大项目制度，强化协调推进责任，坚持每月分析督办，及时解决项目推进中存在的问题。认真执行项目法人负责制、合同管理制、工程监理制、招投标制、工程质量终身负责制，科学组织施工，严格质量管理。加快重点项目征地拆迁进度，保障建设用地需求。协调和引导金融机构加大对重点项目的信贷支持，全年新增贷款90亿元。发挥电力优势，用好丰水期富余电量消纳资金和每年15亿度留存电量政策。强化组织协调，保障煤、电、油、气、运等要素供给。

　　六、优化城镇布局，加快推进新型城镇化

　　新型城镇化既是推动城乡、区域协调发展的重要动力，也是扩大内需、转型升级的战略重点。

　　强化中心城区建设管理。疏解老城、拓展新区，大力实施旧城和棚户区改造，强化姚桥新区建设，推进大兴组团基础设施建设，加快雨城、名山、芦天宝飞地产业园区(四川雅安经济开发区)一体化发展步伐。加快城市道路、桥梁、管网等基础设施建设，启动建设主城区北外环线，加快建设雨名连接线，年底前建成大兴大桥，完成110千米供排水管网建设。改造成雅高速公路姚桥、北郊、多营进出口大道，提升城市门户形象。突出亲水特色，分步打造“一江三河”景观，开工建设周公河左岸道路景观工程，完成姚桥滨河公园建设和北纬30度公园主体工程。强化城市管理，综合整治城市环境。加快智慧城市建设。积极创建省级双拥模范城市、省级文明城市，争创国家级卫生城市。

　　加快县城和重点乡镇建设。加强对县城的规划建设和管理，推进城市道路、桥梁等基础设施，水、电、气等市政公用设施建设。坚持新区拓展和旧城改造并进，突出县城特色，提升县城形象。注重个性特点和历史文化传承，强力推进芦山县龙门乡、飞仙关镇，天全县始阳镇、紫石乡，宝兴县硗碛乡，荥经县龙苍沟镇，雨城区上里镇、中里镇，名山区百丈镇等重点乡镇重建，提升综合承载能力，吸引人口适度集聚。

　　有序推进农业转移人口市民化。深入推进户籍制度改革，全面放开城镇落户限制条件，逐步实施流动人口居住证制度。抓好农民工住房保障制度建设，继续实施“农民工住房保障行动”。推进城镇基本公共服务常住人口全覆盖，完善并落实好农民工参加各项社会保险政策，统一农民工和城镇职工失业保险管理，将农民工及随迁家属纳入社区医疗保障服务体系，保障农民工随迁子女平等接受教育。

　　七、办好民生实事，深入推进社会建设

　　强化社会建设，保障和改善民生，解决人民群众最关心、最直接、最现实的利益问题，是政府的为政之责。

　　大力实施民生工程。扎实推进“九项民生工程”。切实办好20件民生实事：免除6100名经济困难家庭高中学生学费;全面取消县级公立医院药品加成;提供免费婚前体检;新建(改造)养老床位940张，建设28个城乡社区日间照料中心，为18800名困难家庭的失能老人和80周岁以上高龄老人提供居家养老服务;对民族地区城乡“三无”、“五保”对象和孤儿全面提供集中供养;对13712名重度残疾人护理费用给予资助;对初次创业并吸纳3名以上劳动者就业的高校毕业生，一次性给予1万元的创业补贴;将2014年竣工公租房的30%定向出租给农民工;加快推进农村危房改造和地质灾害避险搬迁;对638个行政村公共服务设施运行维护给予补助;完成78个农业技术综合服务站恢复重建;新建19个乡镇计生服务站;恢复重建广播电视站74个、无线广播电视发射台(站)20处;秋季开学前完成179所学校恢复重建任务;修建7040处分散农户污水处理设施;恢复重建乡镇文化站93个，配送社区书屋25个、农家书屋449个;养护农村公路5219.68公里;建设通组公路(林区道路)1000公里;建立健全疾病应急救助制度;中心城区新增公共停车位500个以上。

　　推进社会事业发展。优先发展教育事业，实施教育质量五年振兴计划，培育一批具有社会影响力的品牌学校，推进名师、名校(园)长工程，稳步提升教育质量，改善优秀教师和学生流失现象。大力营造全社会尊师重教的浓厚氛围，积极构建优势互补、有序竞争的教育发展格局。创新机制，引进优质民办学校办学，让优质教育资源惠及更多百姓。规范保教行为，促进学前教育健康发展;全面实施素质教育，加快推进义务教育均衡发展，重点解决农村学校优质资源覆盖问题;大力发展高中阶段教育，稳步提升教育质量;全力推进职业教育发展区规划建设，大力发展职业教育。加大公共卫生服务体系建设，加强全科医生培养，推进医师多点执业，提升医疗服务质量和人民群众健康水平。建成市广电中心并投入使用，加强公共文化基础设施建设和管理，广泛开展群众性文化活动。参加四川省第十二届运动会、第十四届少数民族传统体育运动会，积极承办省级以上体育赛事。启动实施“单独两孩”政策。做好第三次全国经济普查工作。加快发展社会福利、慈善、妇女儿童、残疾人和老龄事业。做好防震减灾、地方志、档案、保密、科协、社会科学、外事、侨务、对台等工作。

　　健全社会保障体系。强化重点群体就业，支持全民创业，推进城乡均等的公共就业创业服务体系建设，动态消除零就业家庭，城镇新增就业16000人。完善各类困难群体救助机制，稳步提高城乡低保和“五保”供养水平。统筹城乡社会保障体系建设，城乡居民养老保险、城镇职工养老保险分别覆盖42.5万人和28万人。健全全民医保体系，扩大城乡医保覆盖面。积极争取国家和省政策扶持，加快贫困地区、革命老区、少数民族地区发展。加大扶贫开发力度，实施“五大扶贫工程”，扶持13500名农村扶贫对象改善生产生活条件。立足库区跨越发展和长治久安，持续抓好库区后续建设、后续管理、后续发展、后续稳定工作。

　　创新社会治理机制。坚持依法治市。强化法制宣传教育，做好“六五”普法工作;加快“平安雅安”建设，加强重点区域、重要目标安全保卫，严厉打击违法犯罪，确保社会治安秩序良好;进一步整合人民调解、行政调解、司法调解力量，推动“大调解”工作向纵深发展;创新群众工作方法，及时化解社会矛盾，维护人民群众合法权益。强化安全生产，坚决遏制重特大安全事故发生。在高危行业全面推广安全生产责任商业保险统保;扎实开展“道路交通安全综合整治攻坚年”行动，加快县乡及以上公路危险路段路侧安保设施建设;继续推进煤矿整顿关闭和煤矿企业兼并重组;推进安全社区建设。深化食品药品安全专项整治，营造放心消费、安全消费的良好环境。加快应急指挥平台体系建设，夯实基层应急管理基础。加强国家安全、民族、宗教、保密、人民防空、国防动员和民兵预备役等工作。

　　八、注重永续发展，加快推进生态文明建设

　　良好的生态资源和生态环境，是实现美丽雅安永续发展的基础。

　　强化生态资源转化，加速提升生态经济效益。以全国生态文明示范工程试点市建设，争取国家生态文明先行示范区、国家主体功能区建设试点示范为抓手，切实将生态文明建设融入经济社会发展全领域、全过程。坚持不懈地推进新型工业化、新型城镇化、农业现代化，静心持久地发展绿色经济、生态经济、循环经济。依托良好的生态资源，针对性开展招商引资和承接产业转移，加快把得天独厚的生态资源优势转化为独具特色的生态经济优势。

　　强化生态基础工程，确保生态资本永续可用。加快修复震损林地、草地、湿地等自然生态系统，强化饮用水源地水源保护设施修复。治理水土流失面积105平方公里。继续实施天然林保护二期、退耕还林、野生动植物保护等生态工程，加快建设天然林保护工程国家级示范市。启动建设芦山围塔漏斗、芦山河、天全河等湿地公园。加快建设大相岭、小相岭大熊猫生态放归基地。力争大相岭自然保护区晋升为省级保护区。

　　持续保护生态环境，确保美丽雅安永续发展。严格遵守水资源开发利用控制、用水效率控制、水功能区纳污能力限制“三条红线”，加强饮用水源管理保护和地下水污染防治。深入开展城乡环境综合治理，不断提升城乡环境质量。继续做好空气质量常规监测。加强环境隐患排查和整治，加大环境污染违法犯罪惩处力度。强化工业企业、机动车尾气、城市扬尘污染监测治理。加大节能减排力度，万元地区生产总值能耗下降和主要污染物排放达到省定目标。

　　九、加快改革开放，全面激发后发追赶的活力

　　改革产生动力，开放促进发展，合作实现共赢。

　　推进重点领域改革。深化行政审批制度改革，公布行政审批事项目录清单;严格落实“两集中、两到位”，行政审批事项在政务服务中心直接办理。加快金融改革创新，支持股份制银行在雅安设立分支机构，积极发展村镇银行、小额贷款和融资性担保公司;组建雅安农村商业银行，完成雅安市商业银行增资扩股。深化国有企业改革，健全现代企业制度，规范和完善企业法人治理结构。深化投融资体制改革，整合、规范投融资平台，推动发行灾后恢复重建债券，适度扩大直接债务融资规模。深化财税体制改革，完善市县(区)分税制财政体制，继续推进增值税改革，完善财政转移支付办法。开展农村土地承包经营权、房屋所有权、集体建设用地使用权、宅基地使用权等确权登记颁证工作，探索建立农村产权交易平台，盘活农村各类产权。探索建立绿色gdp考核体系和生态补偿、碳汇交易等制度。加快公共资源交易领域改革。稳妥推进水电气等资源性产品价格改革。完成市县(区)政府部门机构改革任务。推动劳动社会保障、医疗卫生和文化体制改革，加快教育领域综合改革。

　　提高对外开放水平。抢抓新一轮西部大开发和灾后恢复重建机遇，拓展对外开放的广度和深度，强化外贸出口和外资利用，实现进出口总额7110万美元，实际利用外资2000万美元。强化科学招商、专业招商、务实招商，出台针对性、操作性更强的配套优惠政策，亿元以上投资项目由引进单位或园区负责全程代办相关手续，对投资者居住、生活、医疗、子女入学等提供“保姆式”服务。积极主办灾区招商引资推介会，精心组织参加“中外知名企业四川行”、第十五届西博会等大型招商活动，确保到位市外资金285亿元。今年各县(区)至少引进投资5亿元以上的项目2个。

　　加快推动区域合作。抢抓成都、德阳、绵阳、眉山、乐山、资阳、遂宁、雅安同城化发展机遇，以经济合作为主攻方向，主动融入、配套、服务成都经济圈。加快推进成雅合作园区建设，启动建设雅双产业合作园。加强与藏区合作。提升对周边地区的服务和辐射能力，吸引人口集聚和产业聚集。强化与攀枝花市、凉山州的合作，推动攀西战略资源创新开发试验区建设取得实质性进展。强化与对口援建市在技术、产业、市场等方面的深度合作。加强与四川农业大学的合作，不断完善雅安新型农村科技服务体系。

　　着力壮大县域经济。坚持多予、少取、放活，出台支持县域经济发展的政策，进一步理顺市区之间的责权利关系，完善对县(区)的综合考核办法。支持各县(区)依托资源和传统优势加快发展特色主导产业，壮大县域经济规模。坚持整体联动、错位发展，支持东部片区(雨城、名山、芦天宝飞地产业园区和荥经的一部分)加快发展新型工业和城市经济;南部片区(石棉、汉源)依托水能、矿产和特色农业优势，加快发展清洁载能产业和特色效益农业;西北片区(芦山、天全、宝兴和荥经的一部分)突出生态功能区定位，加快发展生态经济。坚持权利平等、机会平等、规则平等，落实鼓励民营经济发展的政策措施，激发民营企业的活力和创造力。

　　十、强化自身建设，努力构建人民满意的服务型政府

　　强化政府自身建设，坚持为人民服务、对人民负责、受人民监督，主动履职、主动担责、主动作为，是做好政府工作的基础。

　　增强为民执政的意识。深入开展政府系统党的群众路线教育实践活动。始终把群众呼声作为第一信号，真诚听取群众意见，真实反映群众愿望，真心关注群众疾苦。把群众需要作为第一选择，满腔热情、尽心竭力地为群众办实事、做好事、解难事。把群众满意作为第一标准，想问题、办事情、作决策，充分考虑群众意愿，切实维护群众利益。

　　提升依法行政的水平。将依法行政作为政府工作的基本准则，健全重大问题专家咨询、社会公示、风险评估、合法性审查、听证等制度，提升政府决策的科学化水平。主动接受市人大及其常委会的法律监督、工作监督和市政协的民主监督，认真办理人大代表议案、建议、批评、意见和政协委员提案。加强与人大、政协的沟通协调，及时主动报告工作、通报情况，积极支持各民主党派、人民团体开展工作。

　　锤炼务实高效的作风。把求真务实、高效运转作为检验政府工作的标尺，树立真抓实干、埋头苦干的作风，在抓落实上下功夫、求实效。弘扬“5+2”、“白加黑”的奉献精神，增强敢于担当的责任意识，打好灾后恢复重建的“硬仗”，补上建设发展的“欠账”。严格目标管理，加强督查督办，严查不落实的事，处理不落实的人，解决不落实的问题，坚决杜绝“中梗阻”现象，确保令行禁止、政令畅通。坚决防止和纠正政府权力部门化、部门权力个人化、个人权力利益化等问题。

　　恪守廉洁从政的底线。坚决贯彻中央“八项规定”、国务院“约法三章”和省委、省政府“十项规定”，深入整治“四风”问题。强化对重点领域、关键岗位的行政监察和审计监督，对重大项目、专项资金实行全过程跟踪审计，确保敢干事会干事、能干事不出事。坚持用制度管权、管事、管人，从源头上预防和治理腐败。厉行节约、勤俭办事，努力建设一支作风过硬、廉洁奉公的公务员队伍，以廉洁勤政的实际成效取信于民，提升政府的公信力和美誉度。

　　各位代表，新的机遇带来新的希望，新的使命开启新的征程。时代赋予重任，奋斗铸就辉煌。我们将紧密团结在以习近平同志为总书记的党中央周围，在省委、省政府和市委的坚强领导下，求真务实、开拓进取，知难而进、迎难而上，为奋力促进雅安经济社会发展新跨越，加快建设幸福美丽新家园而努力奋斗!
